# 17β-Estradiol affects the innate immune response in common carp

**DOI:** 10.1007/s10695-020-00827-3

**Published:** 2020-06-09

**Authors:** Magdalena Maciuszek, Lukasz Pijanowski, Agnieszka Pekala-Safinska, B. M. Lidy Verburg-van Kemenade, Magdalena Chadzinska

**Affiliations:** 1 Department of Evolutionary Immunology, Institute of Zoology and Biomedical Research, Faculty of Biology, Jagiellonian University, Gronostajowa 9, PL30-387 Krakow, Poland; 2 Department of Fish Diseases, National Veterinary Research Institute, Partyzantow Avenue 57, PL24-100 Pulawy, Poland; 3 Cell Biology and Immunology Group, Wageningen University, P.O. Box 338, 6700AH Wageningen, The Netherlands

**Keywords:** Estrogens, Inflammation, Macrophage polarization, Fish, Bacterial infection

## Abstract

Inflammation is the evolutionary conserved immune response to harmful stimuli such as pathogens or damaged cells. This multistep process acts by removing injurious stimuli and initiating the healing process. Therefore, it must be tightly regulated by cytokines, chemokines, and enzymes, as well as neuroendocrine mediators. In the present work, we studied the immunoregulatory properties of 17β-estradiol (E2) in common carp. We determined the in vitro effects of E2 on the activity/polarization of macrophages and the in vivo effects during *Aeromonas salmonicida*-induced inflammation. In vitro, E2 reduced the lipopolysaccharide (LPS)-stimulated expression of pro- and anti-inflammatory mediator genes but did not change the gene expression of the estrogen receptors and of aromatase CYP19. In contrast, in vivo in the head kidney of *A. salmonicida*-infected fish, E2-treated feeding induced an upregulation of gene expression of pro-inflammatory (*il-12p35* and *cxcb2*) and anti-inflammatory (*arginase 1*, *arginase 2*, *il-10*, and *mmp9*) mediators. Moreover, in infected fish fed with E2-treated food, a higher gene expression of the estrogen receptors and of the aromatase CYP19 was found. Our results demonstrate that estrogens can modulate the carp innate immune response, though the in vitro and in vivo effects of this hormone are contrasting. This implies that estradiol not only induces a direct effect on macrophages but rather exerts immunomodulatory actions through indirect mechanisms involving other cellular targets.

**Electronic supplementary material:**

The online version of this article (10.1007/s10695-020-00827-3) contains supplementary material, which is available to authorized users.

## Introduction

The innate immune response is the first line of defense against pathogens. Although fish are the earliest vertebrates with an adaptive immune response, they still rely mostly on their innate response (Magnadóttir 2006). As in mammals, the professional phagocytes (macrophages and granulocytes) in fish are also the executive cells of the innate response/inflammation. They migrate to the site of inflammation and eliminate pathogens through phagocytosis. Additionally, they produce reactive oxygen species (ROS), enzymes (e.g., lysozyme, neutrophil elastase, and matrix metalloproteinase 9 (MMP9)), and antimicrobial peptides that are all toxic to pathogens. As these latter molecules can be also toxic for host cells and tissues, phagocyte activation has to be tightly regulated by cytokines (Chadzinska et al. 2008b). During the first phase of inflammation, pro-inflammatory cytokines (e.g., interleukins: IL-1β, IL-12, and tumor necrosis factor (TNF-α)) activate phagocytes while their migration is directed by chemokines (e.g., CXCL8/IL8 and CXCb) (van der Aa et al. 2012). In turn, after pathogen eradication, the anti-inflammatory phase takes place when anti-inflammatory cytokines such as IL-10 and IL-1 receptor antagonist (IL-1Ra) orchestrate the resolution of inflammation and tissue repair. The cytokine milieu thus determines the status of cell activation/polarization and thereby the direction of the immune response. Both in mammals and fish, classical pro-inflammatory (M1) polarization of macrophages takes place upon stimulation with pro-inflammatory TNF-α and/or interferon gamma (INF-γ) and in contact with bacterial lipopolysaccharide (LPS). M1 macrophages exhibit a higher expression of pro-inflammatory cytokines (IL-1β, IL-12) and CXC chemokines, and they produce ROS (Arts et al. 2010). This results in a shift of the immune response to a cellular response, involving Th1 lymphocytes (Martinez and Gordon 2014). In contrast, upon stimulation with the anti-inflammatory cytokines IL-10, IL-4, or IL-13, macrophages exhibit an anti-inflammatory M2 phenotype. M2 macrophages are characterized by higher production of anti-inflammatory cytokines (IL-4, IL-10, IL-13) (Montero et al. 2016; Mao et al. 2018). These mechanisms lead to a shift of the immune reaction towards a humoral response with Th2 lymphocytes, immunoregulation, remodeling of the matrix, tissue repair, and silencing of inflammation. M1 and M2 polarization of macrophages is strongly associated with the internal metabolism of l-arginine. Whereas the M1 cells increase their expression of inducible nitric oxide synthase (iNOS), converting l-arginine to N-hydroxy-l-arginine, which results in increased production of nitric oxide (NO), M2 cells increase expression of the arginase, converting l-arginine to urea and l-ornithine. Consequently, polyamines and prolines are formed, which are essential for cell proliferation and tissue repair (Joerink et al. 2006; Rath et al. 2014). Several other proteins such as cysteine-rich angiogenic inducer 61 protein (cyr61), inhibin, and MMP9 are involved in the process of wound healing and tissue regeneration (Bai et al. 2005; Ogawa et al. 2006; Lau 2011; Derlindati et al. 2015). Another group of molecules involved both in pathogen eradication and in host tissue protection during inflammation are liver-derived acute phase proteins (APPs) such as C-reactive protein (CRP), serum amyloid P (SAP), and complement proteins such as C3 (Bayne and Gerwick 2001).

Next to immune-related factors (cytokines, APPs) also hormones are strongly implicated in the regulation of the inflammatory reaction/macrophage polarization (Verburg-van Kemenade et al. 2017). Recently, we found, for example, that both the in vitro and the in vivo polarization of carp macrophages are affected by cortisol (Maciuszek et al. 2019). We also observed that 17β-estradiol (E2) affects the in vitro activity of fish macrophages and that this process is mediated via the membrane estrogen receptor GPR30 (Szwejser et al. 2017a). Moreover, we and others found that fish leukocytes also express nuclear estrogen receptors (ERα and ERβ) (Liarte et al. 2011b; Iwanowicz et al. 2014; Burgos-Aceves et al. 2016; Szwejser et al. 2017a; Paiola et al. 2019). Furthermore, fish leukocytes have been shown to express aromatase - *cyp19a* and *cyp19b* genes encoding aromatase P450, responsible for the conversion of testosterone to 17β-estradiol (Szwejser et al. 2017b). This implicates direct auto- or intra-crine regulation of the immune response by estrogens (Matthews and Gustafsson 2003; Hawkins and Thomas 2004; Katsu et al. 2013; Szwejser et al. 2017a). And indeed, estrogen has been shown to affect the innate response by modulating the production of ROS, NO, and cytokines (Watanuki et al. 2002; Thilagam et al. 2009; Liarte et al. 2011b; Cabas et al. 2012; Szwejser et al. 2017a; Paiola et al. 2019). It also affected phagocytosis and lysozyme activity (Wang and Belosevic 1995; Law et al. 2001; Yamaguchi et al. 2001; Watanuki et al. 2002; Thilagam et al. 2009; Liarte et al. 2011b; Akbary et al. 2013). Furthermore, it was found that E2 may modulate fish susceptibility to infection by altering leukocyte activity. For example, E2 increased the mortality of fish infected with *Trypanosoma danilewskyi* (Wang and Belosevic 1994) while rainbow trout exposed to E2 had higher susceptibility to bacterial infection of *Yersinia ruckeri* and consequently decreased survival rates (Wenger et al. 2011). Moreover, E2 increased susceptibility to viral infection of spring viraemia of carp virus (SVCV) in larvae and in adult zebrafish (López-Muñoz et al. 2015)*.* In contrast, E2 enhanced protection against lice in Atlantic salmon (Krasnov et al. 2015). However, it should be mentioned that the immunoregulatory actions of estrogens vary among fish species, and also with dose, target cell type, or physiological condition (e.g., infection load, stress level, reproductive status) (Szwejser et al. 2017b, c).

In the present work, we examined how E2 may affect the response of carp monocytes/macrophages upon bacterial infection. We studied the in vitro reaction against bacterial lipopolysaccharide. Subsequently, we studied the in vivo inflammatory reaction evoked by an *Aeromonas salmonicida*-induced bacterial infection. For this purpose, we measured the effect on expression of the most important pro- and anti-inflammatory mediators. To better clarify the potential mechanism of E2 action, we moreover studied the expression of the nuclear and membrane estrogen receptors and the aromatase cyp19 enzyme.

## Materials and methods

### Animals

Young, sexually immature individuals of common carp (*Cyprinus carpio* L; line R3xR8) were obtained from the Institute of Ichthyobiology and Aquaculture, Polish Academy of Sciences, Golysz, Poland. Fish used for treatment groups within one experiment were from one breed, and to limit potential differences between experiments, we always used fish aged 9 to 12 months that have a well-developed mature immune system. Studies were conducted in the spring (March/April 2019). Prior to the experiments, fish were adapted for 4 weeks at 21 °C in recirculating tap water at the Institute of Zoology and Biomedical Research in Krakow, Poland. Fish were kept in tanks (volume 375 L, flow rate 4 L/min, density 45 fish/tank and 60 g/L) and fed pelleted dry food (Aller Master, Aller Aqua, Poland) at a daily maintenance rate of 1% of their estimated body weight, in photoperiod 13L:11D. In order to avoid additional stress and/or differences in handling, all samplings were performed by the same person and at the same time of day (at 9:00 a.m.).

All animals were handled in strict accordance with good animal practice as defined by the relevant national and local animal welfare bodies, and procedures were approved by the local ethical committee (2nd Local Institutional Animal Care and Use Committee (IACUC) in Krakow, Poland, license number 291/2017).

### E2 treatment

After acclimation, fish were randomly divided into 2 different treatment groups: non-E2—fed with control food (*n* = 14) or E2—fed with food treated with E2 (20 mg E2/kg food, Sigma-Aldrich, St. Louis, MO, USA) (*n* = 14). The weight of the animals from both groups before the start of the experiment did not differ significantly and was 65.1 g ± 11.75 for control fish and 56.14 g ± 9.18 for experimental animals. The E2 concentration was chosen and the food was prepared as described before (Wenger et al. 2011) from commercially available dry food (Aller Master, Aller Aqua, Poland) which was spiked with E2 diluted in ethanol (99.8%, POCH, Gliwice, Poland) and processed by alcohol evaporation method—E2 (Guerrero 1975) with some modification: food was dried for 2 h at room temperature. Control food (non-E2) was spiked with the same volume of ethanol alone. Fish were fed at a daily maintenance rate of 1% of their estimated body weight for 14 days. After the feeding period, the weight of control and E2-treated fish did not differ significantly and was 72.4 g ± 3.5 and 64.3 g ± 3.3, respectively. Efficiency of E2 treatment was confirmed by measurement of the increased E2 serum levels and of the upregulation of *vitellogenin* gene expression in the liver of fish fed with E2-treated food compared to animals fed with control non-E2 food (Fig. 5).

### Infection

*A. salmonicida* subsp. *salmonicida* from Polish origin was obtained from the Department of Fish Diseases, National Veterinary Research Institute, Pulawy. Bacteria were grown in lysogeny broth (LB) medium for 18 h at 25 °C and centrifuged at 1600*g* for 10 min, and the bacterial pellet reconstituted in sterile PBS (280 mOsM). Optical density was measured at 625 nm, and data were aligned with a previously derived McFarland scale to determine the bacterial concentration. On day 14 of feeding, fish from both E2 and non-E2 groups (*n* = 7) were injected intraperitoneally (i.p.) with a non-lethal dose of *A. salmonicida* (4 × 10^8^ bacteria in 250 μL PBS per fish) as described previously (Falco et al. 2012). Fish were sacrificed at 24 and 96 h post-injection (hpi). Fish were anesthetized with tricaine methane sulfonate (TMS; Sigma-Aldrich, St. Louis, MO, USA; 0.2 g/L) buffered with NaHCO_3_ (POCH, Gliwice, Poland; 0.4 g/L).

### Serum hormone level

Fish were bled immediately post anesthesia through puncture of the caudal vein using a needle attached to a 5-mL syringe. The samples were taken midline, just posterior of the anal fin. Each time, approximately 5 mL of blood was removed from the caudal vein into the syringe. Blood was collected in covered test tubes and allowed to clot overnight at 4 °C. Blood clots were removed by centrifuging at 3000×*g* for 30 min, and serum was collected and stored at − 20 °C for future use. Estradiol level was determined using the commercial kit DRG (Marburg, Germany; range 10.6–2000 pg/mL, sensitivity 10.60 pg/mL). On the day of the assay, blood serum samples were thawed and used according to the manufacturer’s protocol. All standards and samples from every individual fish were analyzed in duplicate, in the same batch.

### Organ and cell isolation

After the bleeding, peritoneal leukocytes (PTL) were taken by flushing the peritoneal cavity with 2 mL of sterile PBS (280 mOsM) with heparin (Leo Pharmaceutical Products Ltd., Weesp, the Netherlands) as described previously (Chadzinska et al. 2008b). To determine the composition of peritoneal leukocytes, the phagocytes (mononuclear and polymorphonuclear leukocytes) and lymphocytes were counted in a hemocytometer after Türk staining (0.01% (w/v) crystal violet (Sigma-Aldrich, St. Louis, MO, USA) in 3% (v/v) acetic acid (POCH, Gliwice, Poland) which allows distinction of the smaller, spherical lymphocytes with a round nucleus and a small non-granular cytoplasm from the bigger, granular phagocytes with irregular-shaped nucleus (Chadzinska et al. 2008b).

Organs (head kidney and liver) were carefully removed and immediately transferred to fix RNA buffer (Eurex, Gdansk, Poland) and kept at − 20 °C for further analysis.

### In vitro cell isolation and stimulation

Monocyte/macrophage suspensions were obtained by passing the head kidney tissue from intact fish through a 100-μm nylon mesh with carp RPMI (cRPMI 1640, Invitrogen, Carlsbad, CA, adjusted to carp osmolarity of 270 mOsm/kg with distilled water) containing heparin (Leo Pharmaceutical Products Ltd., Weesp, the Netherlands) and washed once as described previously (Maciuszek et al. 2019). This cell suspension was layered on a discontinuous Percoll gradient (1.020, 1.060, 1.070, and 1.083 g/cm^3^) to retrieve enriched populations of monocytes/macrophages (1.060 g/cm^3^) and centrifuged for 30 min at 800×*g* with the brake disengaged (Verburg-van Kemenade et al. 1994). Cell fractions were collected and washed. To verify the purity of the isolated cell populations, the monocyte/macrophage-enriched cell population was stained with neutral red (NR) (0.1 mg/mL; 3 min, at RT; Sigma-Aldrich, USA) or with Türk’s solution for 3 min at RT and analyzed in a hemocytometer under a light microscope. Additionally, with a FACScalibur flow cytometer (BD Biosciences), 30,000 threshold events per sample were analyzed for their forward scatter (FSC) (for cell size) and sideward scatter (SSC) (cell complexity) profiles. Data were analyzed using WinMDI 2.9 software (Joe Trotter, http://facs.scripps.edu).

Cell fractions obtained from the head kidney contained an abundance of macrophages (48.86% ± 1.38) (Maciuszek et al. 2019).

The monocyte/macrophage-enriched suspensions were resuspended in carp cRPMI++ (cRPMI supplemented with 0.5% (v/v) pooled carp serum with antibiotics (1%(v/v) l-glutamine (Sigma-Aldrich, St. Louis, MO, USA), 1% (v/v) penicillin G (Sigma-Aldrich, St. Louis, MO, USA) and 1% (v/v) streptomycin sulfate (Sigma-Aldrich, St. Louis, MO, USA)) to a density of 10 million cells per mL. The E2 level in cRPMI++ medium was measured as described previously (Szwejser et al. 2017b) and was below detection level.

To determine gene expression, monocytes/macrophages were seeded in 24-well plate cell culture plates (Nest Biotech Co, Wuxi, China) at 27 °C, 5% CO_2_. The cells were treated either with 1 μM E2 (Sigma-Aldrich, St. Louis, MO, USA) or 30 μg/mL LPS (*Escherichia coli* serotype O55: B5, Sigma-Aldrich, St. Louis, MO, USA; L2880) or their combination (1 μM E2 + 30 μg/mL LPS). E2 and LPS concentrations were chosen based on our previous studies (Szwejser et al. 2017a).

The E2 stock (20 μg/mL) was prepared in 100% ethanol and further diluted in culture medium. Control cells (CTR) were treated with the same volume of vehicle diluted in culture medium. After 6 h of stimulation, the cells were resuspended in 350 μL RL buffer (Eurex, Gdansk, Poland) with 1% (v/v) β2-mercaptoethanol (Sigma-Aldrich, St. Louis, MO, USA) and kept in − 80 °C for further analyses.

To determine cell activity (arginase activity and NO production), monocytes/macrophages were seeded in 96-well plate cell culture plates (Nest Biotech Co, Wuxi, China) at 27 °C, 5% CO_2_ for 24 h and stimulated with E2, LPS, or E2 + LPS. The in vitro experiments were performed 3 times independently, and each time, monocyte/macrophage primary cultures were made from 1 or 2 fish.

### Arginase activity

Arginase activity was measured as described by Corraliza et al. (1994). Cells were lysed with 50 μL of 0.1% (v/v) Triton X-100 containing 5 μg of pepstatin (Sigma-Aldrich, St. Louis, MO, USA), 5 μg of aprotinin (Sigma-Aldrich, St. Louis, MO, USA), and 5 μg of antipain (Sigma-Aldrich, St. Louis, MO, USA), at room temperature for 30 min. After incubation, 35 μL of 10 mM MnCl_2_ (Sigma-Aldrich, St. Louis, MO, USA) and 50 mM Tris-HCl (pH 7.5) (Tris - Biorad, USA; HCl–POCh, Gliwice, Poland) were added to each sample and the mixture was incubated for 20 min at 55 °C. To 50 μL of this activated lysate, 50 μL of 0.5 M l-arginine (pH 9.7) (Sigma-Aldrich, St. Louis, MO, USA) was added and incubated for 1 h at 37 °C. The reaction was stopped by adding 400 μL of an acid mixture containing H_2_SO_4_ (POCh, Gliwice, Poland), H_3_PO_4_ (Chempur, Piekary Slaskie, Polska), and H_2_O (1:3:7). In the next step, 25 μL of 9% (v/v) α-isonitrosopropiophenone (Sigma-Aldrich, St. Louis, MO, USA) in 100% ethanol (99.8%, POCH, Gliwice, Poland) was added to each sample. Samples were incubated for 45 min at 100 °C. After 10 min cooling in the dark, the OD was read at 540 nm, and the arginase activity was calculated by comparison with a urea standard curve (0–6.66 mM).

### Nitric oxide production

Nitrite/nitrate production, an indicator of nitric oxide synthesis, was measured in cell culture supernatants as described previously (Chadzinska et al. 2009). Following 24 h incubation, 100 μL cell culture supernatant was added to 50 μL 1% (w/v) sulfanilamide in 2.5% (v/v) phosphoric acid and 50 μL of 0.1% (w/v) N-naphthyl-ethylene-diamine in 2.5% (v/v) phosphoric acid (all from Sigma-Aldrich, St. Louis, MO, USA). The OD reading at 540 nm (with 690 nm as a reference) was taken using the cRPMI++ medium as blank. Nitrite concentration was calculated by comparison with a sodium nitrite standard curve (0–10 μM).

### Gene expression

#### RNA isolation

RNA was isolated from cells and tissues with a GeneMATRIX Universal RNA Purification Kit (Eurex, Gdansk, Poland) according to the manufacturer’s protocol. Final elution was carried out in 30 μL of nuclease-free water, to maximize the concentration of RNA. Before proceeding with further analyses, RNA was quantified, and its integrity checked (Tecan Spark NanoQuant Plate^TM^). Samples were stored at − 80 °C.

#### cDNA synthesis

For each sample, a non-reverse transcriptase (non-RT) control was included. The cDNA synthesis was performed with High-Capacity cDNA Reverse Transcription Kits (Applied Biosystems, Waltham, MA, USA) according to the manufacturer’s protocol. Briefly, 1 μg of total RNA was added to 10 μL RT master mix containing 2 μL 10X RT buffer, 0.8 μL 25XdNTP mix (100 mM), 2 μL 10XRT random primers, 1 μL MultiSribe™ reverse transcriptase, and 4.2 μL of nuclease-free water. The samples were then placed into the thermal cycler (Ditabis AG, Pforzheim, Germany; 25 °C at 10 min, 37 °C at 120 min, 85 °C at 5 min followed by rapid cooling to 4 °C). The samples were set at 100 μL with nuclease-free water and stored at − 20 °C until further use.

#### Real-time quantitative PCR

Carp-specific primers (5′–3′) were used for detection of immune-related (*inos*, *il-1β*, *il-12p35*, *cxcl8_l1*, *cxcl8_l2*, *cxcb1*, *cxcb2*, *cxcr1–3*, *c3*, *crp1*, *crp2*, *il-10*, *arginase 1*, *arginase 2*, *mmp9*, *cyr61*, *inhba*, *tgm2*) and endocrine-related (*erα*, *erβ*, *gpr30*, *cyp19a*, *cyp19b*, *vitellogenin*) genes. The 40S ribosomal protein s11 (*40s11*) gene served as an internal standard. Accession numbers and primer sequences are listed in Table 1.

**Table 1:** Primers used for quantitative real-time PCR analysis

Gene	Primer forward (5′–3′)	Primer reverse (5′–3′)	Acc. no.	μM
*40s11*	CCGTGGGTGACATCGTTACA	TCAGGACATTGAACCTCACTGTCT	AB012087	1
*inos*	AACAGGTCTGAAAGGGAATCCA	CATTATCTCTCATGTCCAGAGTCTCTTCT	AJ242906	1
*il-1β*	AAGGAGGCCAGTGGCTCTGT	CCTGAAGAAGAGGAGGCTGTCA	AJ245635	1
*il-12p35*	TGCTTCTCTGTCTCTGTGATGGA	CACAGCTGCAGTCGTTCTTGA	AJ480354	1
*cxcl8_l1*	CTGGGATTCCTGACCATTGGT	GTTGGCTCTCTGTTTCAATGCA	AJ421443	1.125
*cxcl8_l2*	TCACTTCACTGGTGTTGCTC	GGAATTGCTGGCTCTGAATG	AB470924	1.125
*cxcb1*	GGGCAGGTGTTTTTGTGTTGA	AAGAGCGACTTGCGGGTATG	AB082985	1.125
*cxcb2*	AGGCAGGTGCTTCTGTGCTGACA	TTCATGCATTTCCGCTCTGCGCT	JN104598	1.125
*cxcr1*	GCAAATTGGTTAGCCTGGTGA	AGGCGACTCCACTGCACAA	AB010468	1.125
*cxcr2*	TATGTGCAAACTGATTTCAGGCTTAC	GCACACACTATACCAACCAGATGG	AB010713	1.125
*cxcr3*	TGTCAATGACCCCAAGCATCTGC	CACTCTGTCACGCCACTCGTAGG	HE584636	1.125
*arginase 1*	TGAGGAGCTTCAGCGGATTAC	CCTATTATTCCCACGCAGTGATG	AJ871264	1
*arginase 2*	GGAGACCTGGCCTTCAAGCATCT	CTGATTGGCACGTCCAACT	AJ618955	1
*il-10*	CGCCAGCATAAAGAACTCGT	TGCCAAATACTGCTCGATGT	AB110780	1
*mmp9*	ATGGGAAAGATGGACTGCTG	TCAAACAGGAAGGGGAAGTG	AB057407	2.25
*cyr61*	AGTAGTCGGCTGCGTC	AATGCGGTTGTCATCATC	XM_019079046.1	2
*inhba*	TCCATCAAAGTCCAGCCTCT	CACCCTCACTGTCACCTTCC	XM_019110494.1	1
*tgm2*	GCCTGGTATTTTGGACAGT	GCACTCAGCACTCTTGT	XM_019104041.1	1
*vitellogenin*	TGAAATTCTTCAGACCCCCATT	CATGGCCACATTGTTTGCA	AF414432.1	2
*c3*	GGTTATCAAGGGGAGTTGAGCTAT	TGCTGCTTTGGGTGGATGGGT	AB016215	1
*crp1*	AGCAATGCAACATTTTTCCGTC	ACTTGCGTCAAAGCCACCCAC	JQ010977	2
*crp2*	GATGCTGCAGCATTTTTCAGTC	CTCCGCATCAAAGTTGCTCAAAT	JQ010978	2
*erα*	ACTGCCCACAAACTCTCACC	TGGGAACTCATAGGCTCCAT	BAF99812.1	1
*erβ*	CCAGGTCCATTTGTTGGAGT	TGAGGTCTGGGGAGAAAATG	BAB91218.1	1
*gpr30*	CGACTCTGCTTCCTTTCACC	GATCGTCACCTCAAGCCATT	XM_019067213.1	1
*cyp19a*	GGTGCCCAAGACAATGTATATGG	TTGTCCGATGGTGTCTGATGG	DQ534411.1	1
*cyp19b*	ATGATGGAGCAGGTCGTCAAG	TCAACGCCATCAACGTTACC	EU375456.1	1

For RQ-PCR, 4 μL cDNA and forward and reverse primers (2 μL each) were added to 7 μL SYBR®Select Master Mix (Applied Biosystems, Waltham, MA, USA). RQ-PCR (2 min at 50 °C, 2 min at 95 °C, 40 cycles of 15 s at 95 °C, 60 s at 60 °C) was carried out with a Rotor-Gene Q (Qiagen, Hilden, Germany). Following each run, melt curves were collected by detecting fluorescence from 60 to 90 °C at 1 °C intervals.

Constitutive expression was rendered as a ratio of target gene vs. reference gene (40S ribosomal protein s11 gene) and was calculated according to the following equation:

$$ \mathrm{ratio}=\frac{{\left({E}_{\mathrm{reference}}\right)}^{Ct_{\mathrm{reference}}}}{{\left({E}_{\mathrm{target}}\right)}^{Ct_{\mathrm{target}}}} $$

Changes in gene expression upon E2 treatment and/or cell stimulation were rendered as a ratio of target gene vs. reference gene (40S ribosomal protein s11 gene) relative to their expression in control samples according to the following equation:

$$ \mathrm{ratio}=\frac{{\left({E}_{\mathrm{target}}\right)}^{\Delta  Ct}{\mathrm{target}}^{\left(\mathrm{control}-\mathrm{sample}\right)}}{{\left({E}_{\mathrm{reference}}\right)}^{\Delta  Ct}{\mathrm{reference}}^{\left(\mathrm{control}-\mathrm{sample}\right)}} $$

where *E* is the amplification efficiency and *Ct* is the number of PCR cycles needed for the signal to exceed a predetermined threshold value (Pfaffl 2001).

### Statistical analysis

Data were expressed as mean and standard error (S.E.). Bartlett’s test was performed to ensure the suitability of the data for parametric significance tests. Significant differences between infected groups were assessed by one-way ANOVA followed by post hoc Tukey’s test in case of normally distributed data, or with the non-parametric Kruskal-Wallis test followed by Dunn’s test for data that were not normally distributed. The differences were considered statistically significant at *p* < 0.05.

## Results

### In vitro effects of E2

**Fig. 1:**
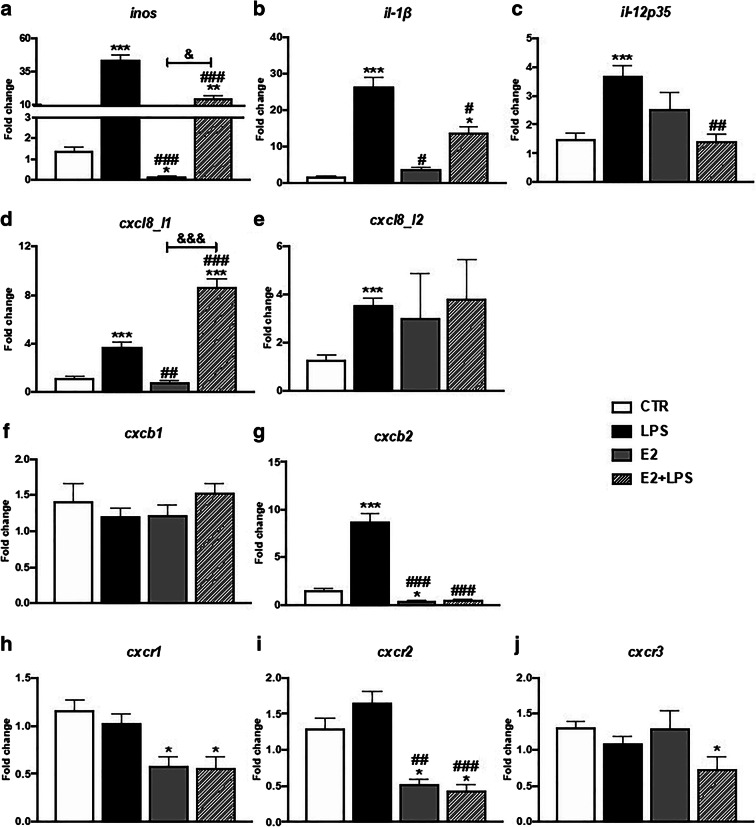
In vitro effects of 17β-estradiol on the gene expression of pro-inflammatory mediators (**a**–**c**), CXC chemokines (**d**–**g**), and their receptors (**h**–**j**) in the head kidney monocytes/macrophages. Cells were in vitro treated for 6 h with lipopolysaccharide (LPS, 30 μg/mL), 17β-estradiol (E2, 1 μM), or their combination (E2 + LPS). Changes in gene expression are shown as *x*-fold increase compared to unstimulated, treated with culture medium cells (CTR), and they were standardized for the housekeeping gene 40S ribosomal protein s11. Averages and S.E. (*n* = 4–5). Stars (*) indicate statistically significant differences between control (CTR) and treated cells (E2, LPS, and E2 + LPS) (*p ≤ 0.05, **p ≤ 0.001, ***p ≤ 0.0001), number signs (#) indicate statistically significant differences between LPS-treated cells (LPS) and E2- or E2 + LPS-treated cells (#*p* ≤ 0.05, ##*p* ≤ 0.001, ###*p* ≤ 0.0001), and ampersands (&) indicate statistically significant differences between E2- and E2 + LPS-treated cells (&*p* ≤ 0.05)

**Fig. 2:**
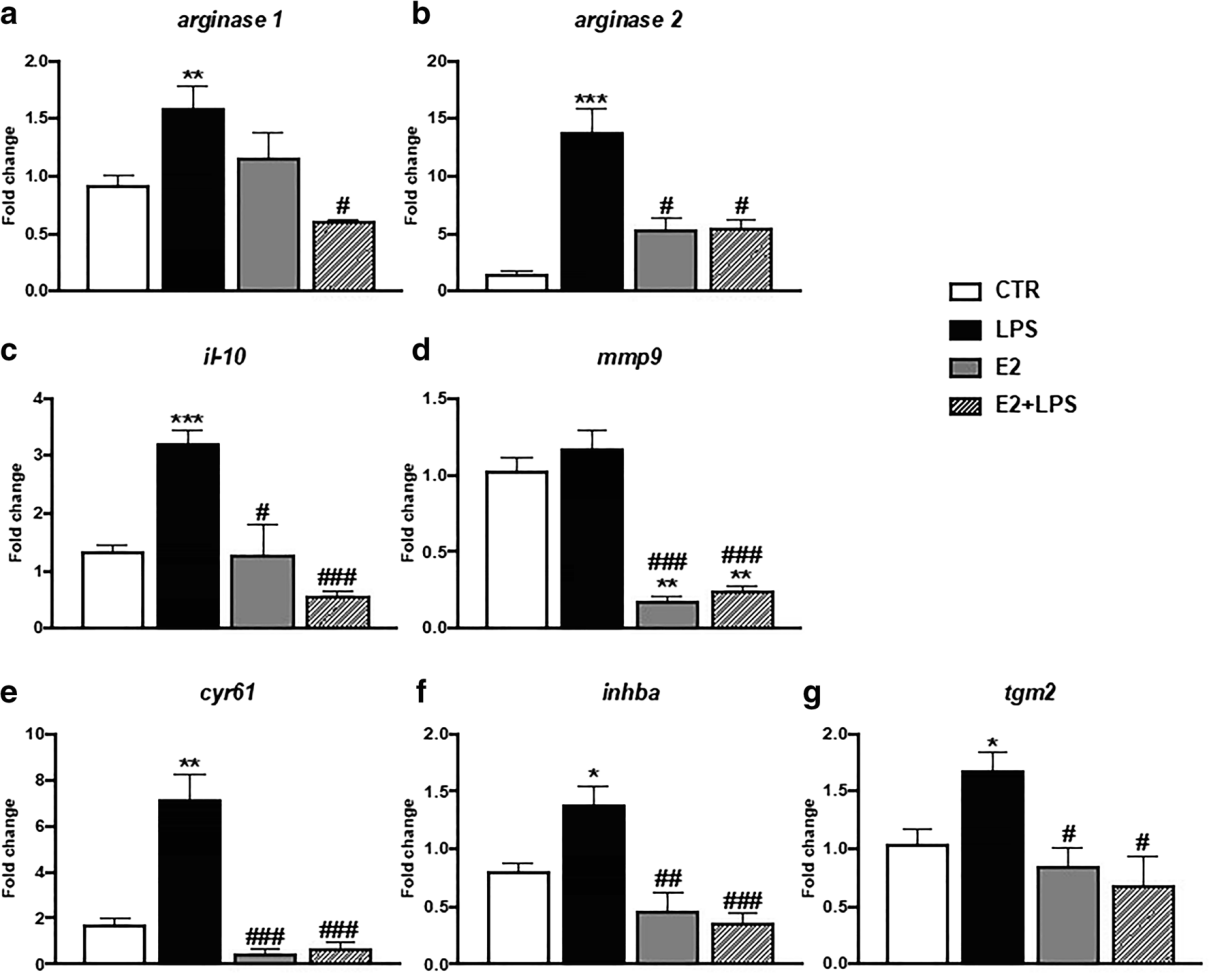
In vitro effects of 17β-estradiol on the gene expression of anti-inflammatory mediators in the head kidney monocytes/macrophages. Cells were in vitro treated for 6 h with lipopolysaccharide (LPS, 30 μg/mL), 17β-estradiol (E2, 1 μM), or their combination (E2 + LPS). Changes in gene expression are shown as *x*-fold increase compared unstimulated, treated with culture medium cells (CTR) and were standardized for the housekeeping gene 40S ribosomal protein s11. Averages and S.E. (*n* = 4–5). Stars (*) indicate statistically significant differences between control (CTR) and treated cells (E2, LPS, and E2 + LPS) (*p ≤ 0.05, **p ≤ 0.001, ***p ≤ 0.0001), number signs (#) indicate statistically significant differences between LPS-treated cells (LPS) and E2- or E2 + LPS-treated cells (#*p* ≤ 0.05, ##*p* ≤ 0.001, ###*p* ≤ 0.0001)

Consistent with previous research, in monocytes/macrophages in vitro, LPS stimulated gene expression of pro-inflammatory mediators: *inos*, *il-1β*, *il-12p35*, *cxcl8_l1*, *cxcl8_l2*, and *cxcb2*) (Fig. 1 and Table 1S) as well as anti-inflammatory: *arginase 1*, *arginase 2*, *il-10*, and *cyr61*, *inhba*, and *tgm2* (Fig. 2 and Table 1S).

E2 alone downregulated gene expression of *inos*, *cxcb2*, *cxcr1*, *cxcr2* (Fig. 1 and Table 1S), and *mmp9* (Fig. 2d and Table 1S).

LPS + E2-treated cells showed a downregulated gene expression of *inos*, *il-1β*, *il-12p35*, *cxcb2*, and *cxcr2* (Fig. 1 and Table 1S) as well as *arginase 1* and *2*, *il-10*, *mmp9*, *cyr61*, *inhba*, and *tgm2* (Fig. 2 and Table 1S) compared to cells treated with LPS only.

Only the *cxcl8_l1* gene expression was synergistically upregulated upon treatment with LPS + E2 (Fig. 1d and Table 1S).

**Fig. 3:**
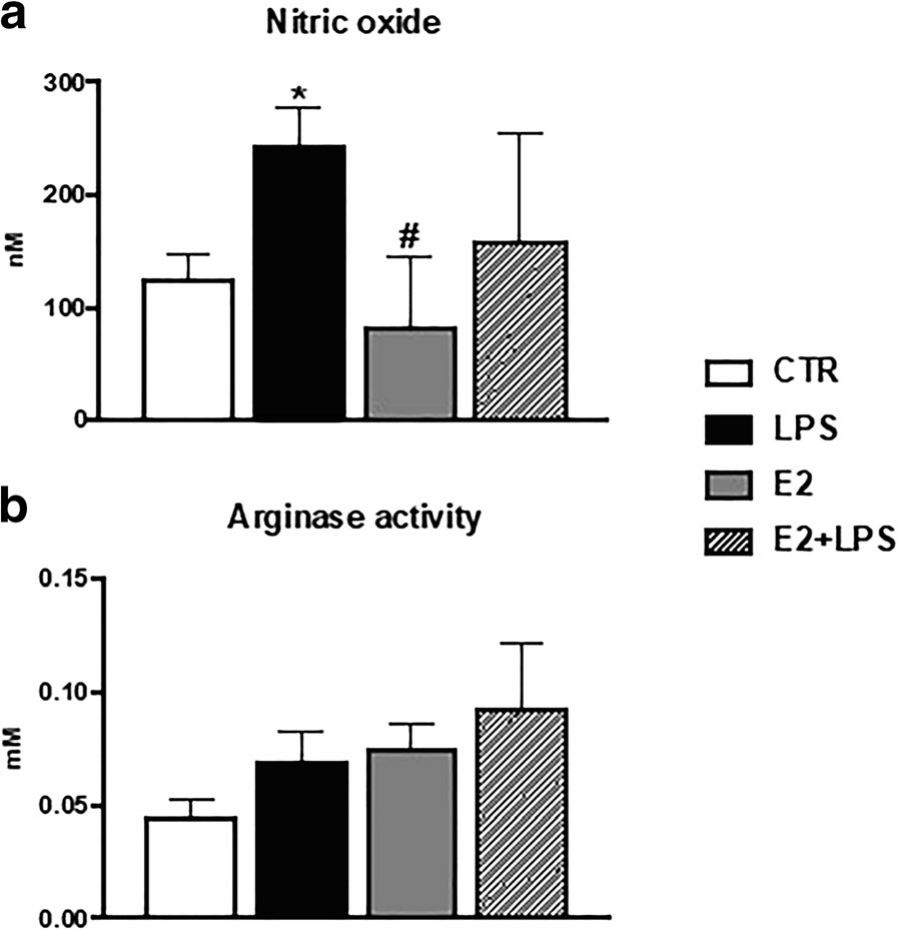
In vitro effects of 17β-estradiol on the production of nitric oxide (**a**) and on the arginase activity (**b**) in the head kidney monocytes/macrophages. Cells were in vitro treated for 24 h with either lipopolysaccharide (LPS, 30 μg/mL) or 17β-estradiol (E2, 1 μM) or their combination (E2 + LPS) or were treated with culture medium (CTR). Averages and S.E. (*n* = 4–5). Stars (*) indicate statistically significant differences between control (CTR) and treated cells (E2, LPS, and E2 + LPS) (*p ≤ 0.05), number signs (#) indicate statistically significant differences between LPS-treated cells (LPS) and E2- or E2 + LPS-treated cells (#*p* ≤ 0.05)

Moreover, upon in vitro LPS treatment, increased levels of NO were found in supernatants from monocytes/macrophages, while E2 did not change the NO production in unstimulated nor in LPS-stimulated cells. Moreover, neither LPS nor E2 affected the activity of arginase (Fig. 3).

**Fig. 4:**
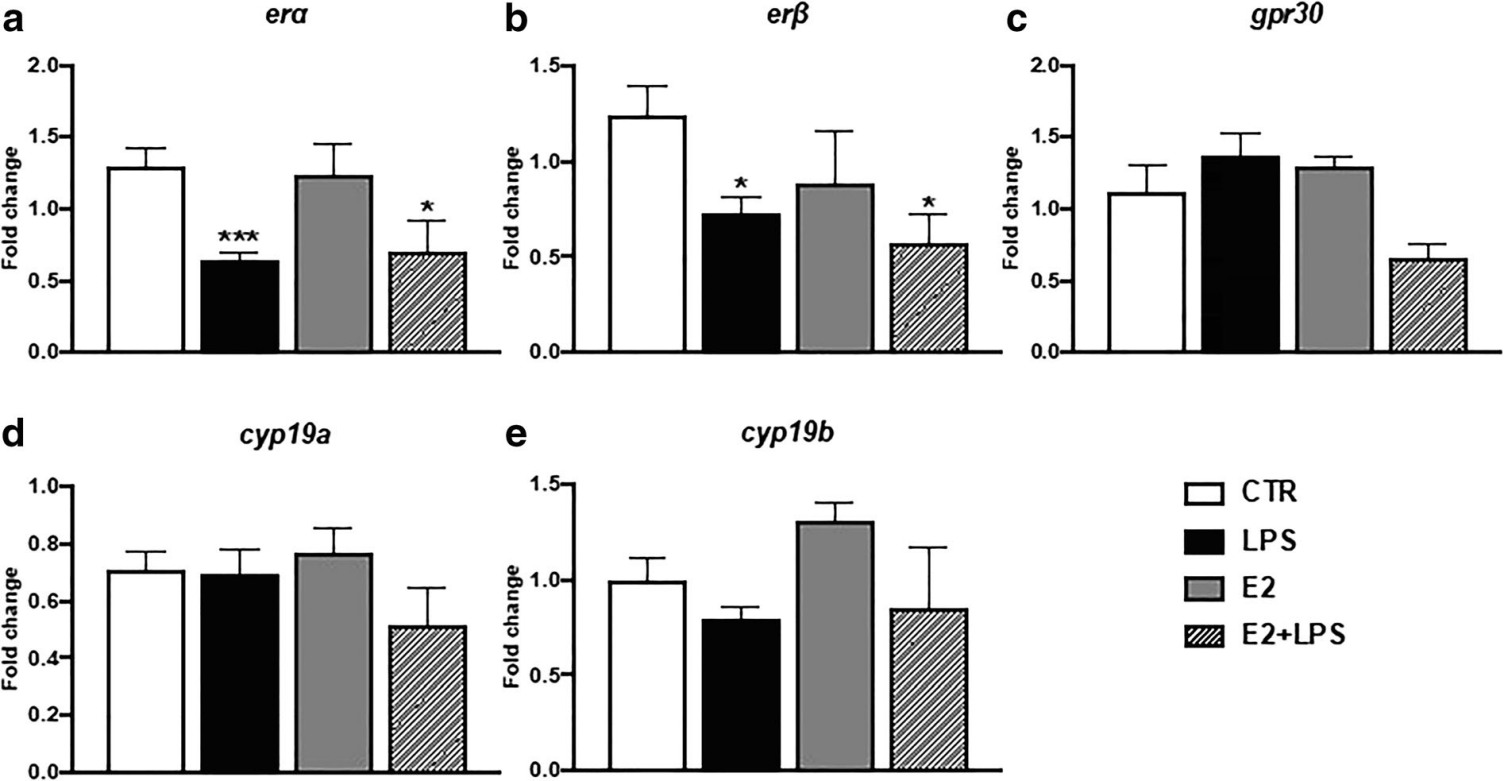
In vitro effects of 17β-estradiol on the gene expression of estrogen receptors (**a**–**c**) and aromatase CYP19 (**d**–**e**) in the head kidney monocytes/macrophages. Cells were in vitro treated for 6 h with lipopolysaccharide (LPS, 30 μg/mL), 17β-estradiol (E2, 1 μM), or their combination (E2 + LPS). Changes in gene expression are shown as *x*-fold increase compared to unstimulated, treated with culture medium, cells (CTR) and were standardized for the housekeeping gene 40S ribosomal protein s11. Averages and S.E. (*n* = 4–5). Stars (*) indicate statistically significant differences between control (CTR) and treated cells (E2, LPS, and E2 + LPS) (*p ≤ 0.05, ***p ≤ 0.0001)

In monocytes/macrophages in vitro, LPS decreased the gene expression of *erα* and *erβ* (Fig. 4a, b and Table 1S), while both E2 and LPS + E2 did not affect the expression of the *erα*, *erβ*, *gpr30*, and *cyp19* genes in these cells (Fig. 4 and Table 1S).

### In vivo effects of E2

#### Level of hormone and vitellogenin gene expression

**Fig. 5:**
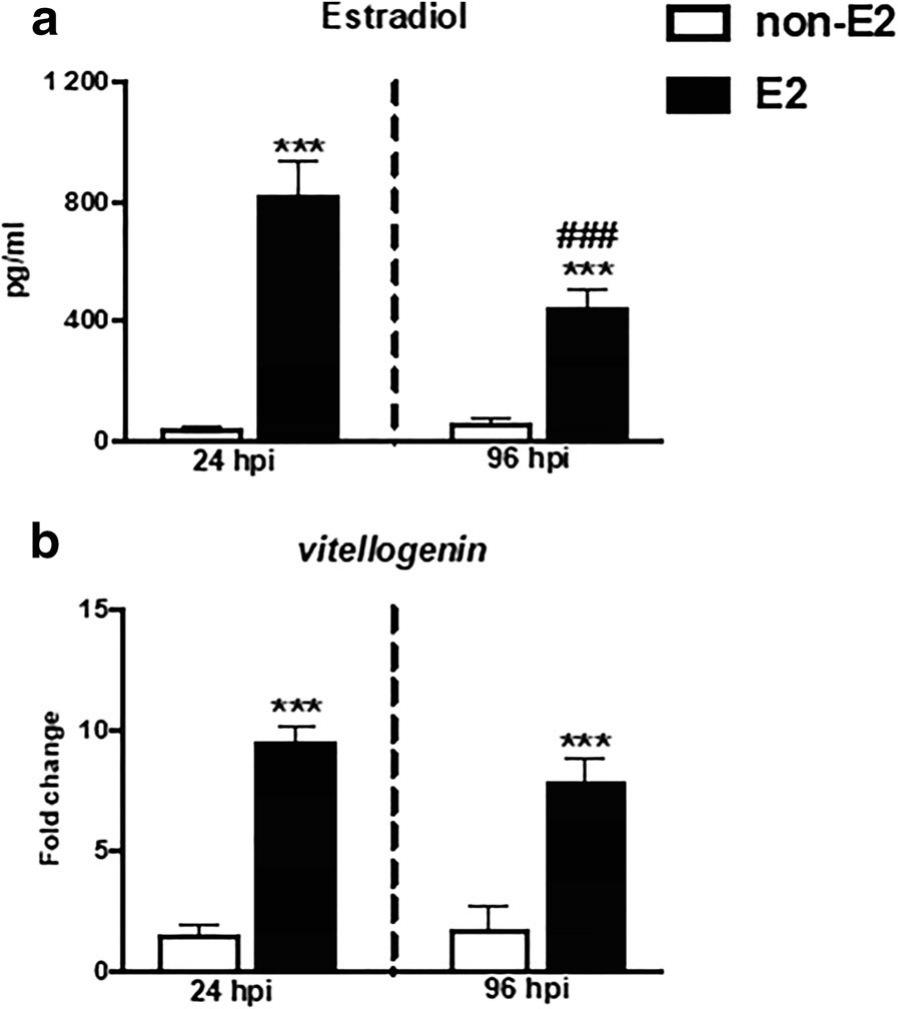
In vivo effects on the level of 17β-estradiol in blood plasma (**a**) and gene expression of *vitellogenin* in liver (**b**). Fish were fed for 14 days with control food (non-E2) or food treated with 17β-estradiol (E2, 20 mg/kg food). On day 14 of E2 feeding, fish were injected i.p. with *A. salmonicida* (4 × 10^8^ bacteria in 250 μL PBS per fish). At 24 and 96 h post-infection (hpi), the blood (to measure E2 level) and livers (to measure *vitellogenin* gene expression) were collected. Averages and S.E. (*n* = 7). Changes in gene expression are shown as *x*-fold increase compared to the control group (CTR) and standardized for the housekeeping gene 40S ribosomal protein s11. Stars (*) indicate statistically significant differences between control (CTR) and E2-treated animals (***p ≤ 0.0001); number signs (#) indicate statistically significant differences between time points of infection (24 vs 96 hpi) (###*p* ≤ 0.0001)

In fish fed with E2-treated food, both at 24 and 96 hpi, a significantly higher level of E2 was found in blood serum and an upregulation of the *vitellogenin* gene expression was observed in the liver, compared to the levels in animals fed with non-E2 food (Fig. 5 and Table 2S). The level of E2 in E2-treated fish was higher at 24 hpi than at 96 hpi (Fig. 5a).

#### Number and composition of inflammatory leukocytes

**Fig. 6:**
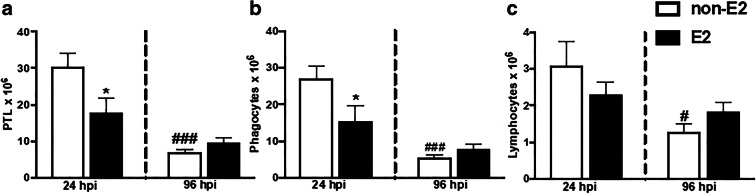
In vivo effects of 17β-estradiol on the number and composition of peritoneal leukocytes. Fish were fed for 14 days with control food (non-E2) or food treated with 17β-estradiol (E2, 20 mg/kg food). On day 14 of E2 feeding, fish were injected i.p. with *A. salmonicida* (4 × 10^8^ bacteria in 250 μL PBS per fish). At 24 and 96 h post-infection (hpi), peritoneal leukocytes (PTL) were collected and their number and composition (number of phagocytes and lymphocyte) were analyzed. Averages and S.E. (*n* = 7). Stars (*) indicate statistically significant differences between control (CTR) and E2-treated animals (*p ≤ 0.05); number signs (#) indicate statistically significant differences between time points of infection (24 vs 96 hpi) (#*p* ≤ 0.05, ###*p* ≤ 0.0001)

In control fish, the total number of peritoneal leukocytes (Fig. 6a) and from these the number of phagocytic cells (Fig. 6b) and lymphocytes (Fig. 6c) were lower at 96 hpi than at 24 hpi. At 24 hpi, E2 decreased the total number of peritoneal leukocytes (Fig. 6a) and the number of phagocytes (Fig. 6b).

#### Gene expression of pro- and anti-inflammatory mediators

**Fig. 7:**
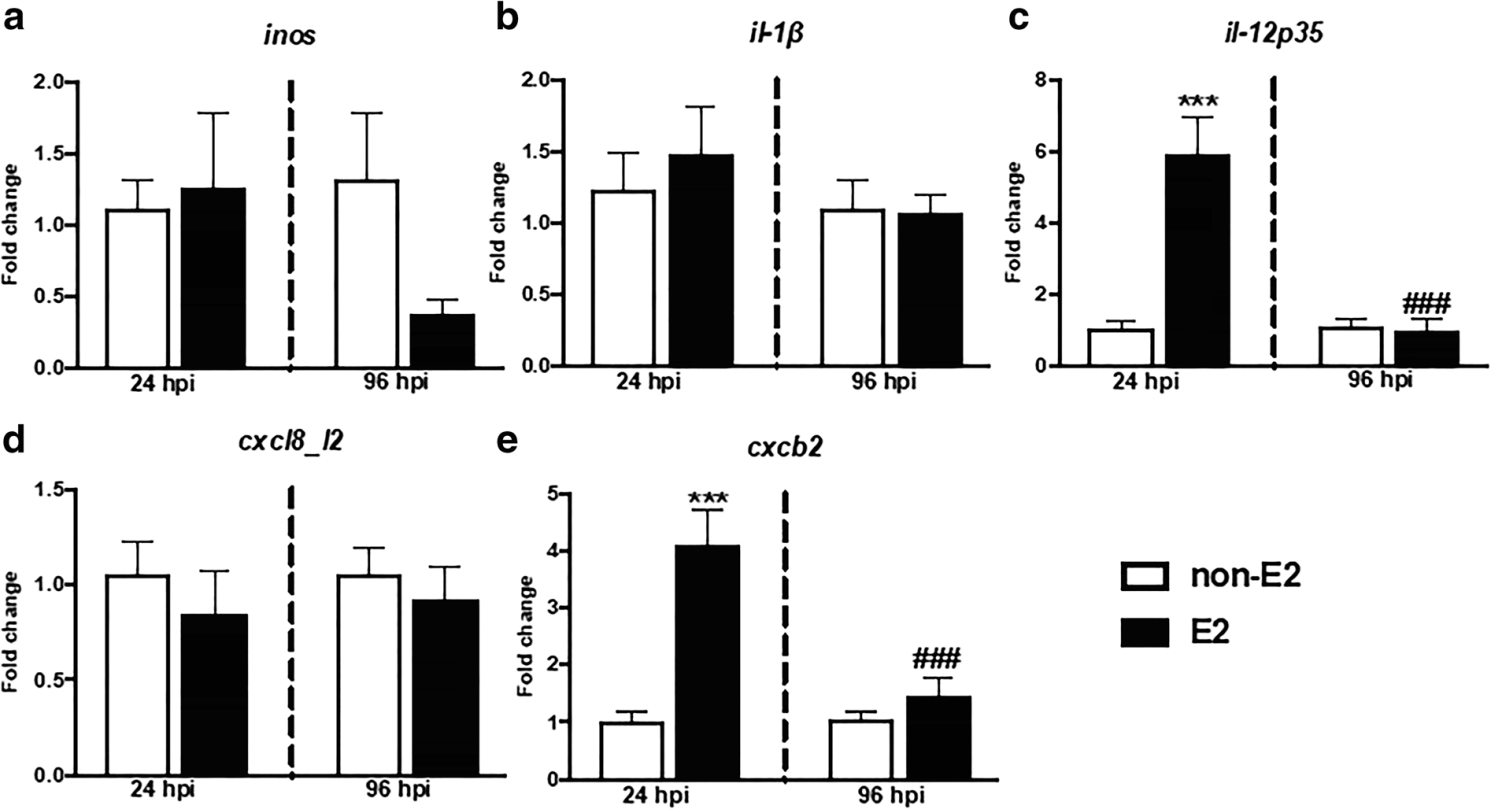
In vivo effects of 17β-estradiol on the gene expression of pro-inflammatory mediators (**a**–**c**) and CXC chemokines (**d**–**e**) in the head kidney. Fish were fed for 14 days with control food (non-E2) or food treated with 17β-estradiol (E2, 20 mg/ kg food). On day 14 of E2 feeding, fish were injected i.p. with *A. salmonicida* (4 × 10^8^ bacteria in 250 μL PBS per fish). At 24 and 96 h post-infection (hpi), the head kidneys were collected, and gene expression was measured. Changes in gene expression are shown as *x*-fold increase compared to control group (non-E2) and standardized for the housekeeping gene 40S ribosomal protein s11. Averages and S.E. (*n* = 7). Stars (*) indicate statistically significant differences between control (CTR) and E2-treated animals (***p ≤ 0.0001), number signs (#) indicate statistically significant differences between time points of infection (24 vs 96 hpi) (###*p* ≤ 0.0001)

**Fig. 8:**
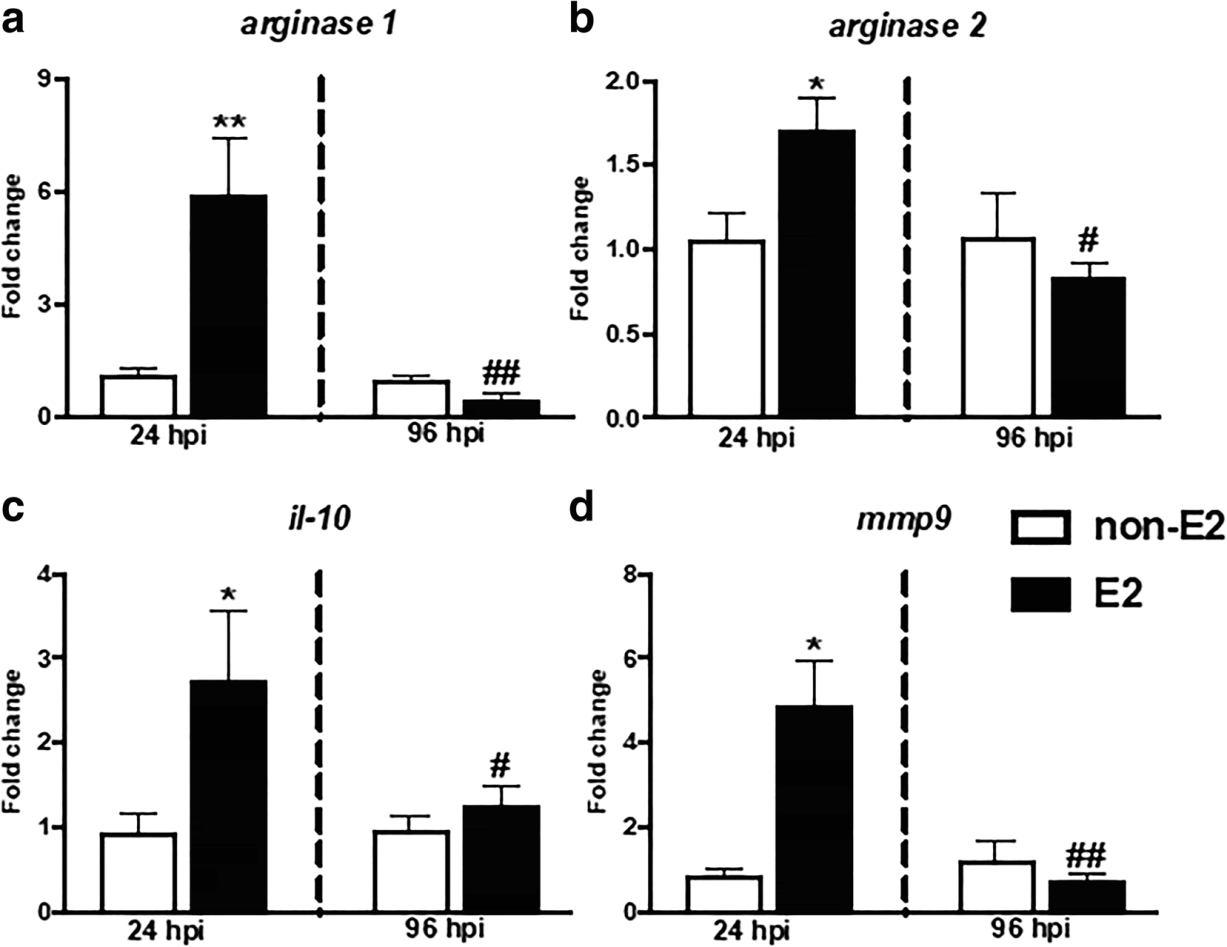
In vivo effects of 17β-estradiol on the gene expression of anti-inflammatory mediators in the head kidney. Fish were fed for 14 days with control food (non-E2) or food treated with 17β-estradiol (E2, 20 mg/ kg food). On day 14 of E2 feeding, fish were injected i.p. with *A. salmonicida* (4 × 10^8^ bacteria in 250 μL PBS per fish). At 24 and 96 h post-infection (hpi), the head kidneys were collected, and gene expression was measured. Changes in gene expression are shown as *x*-fold increase compared to control group (non-E2) and standardized for the housekeeping gene 40S ribosomal protein s11. Averages and S.E. (*n* = 7). Stars (*) indicate statistically significant differences between control (CTR) and E2-treated animals (*p ≤ 0.05, **p ≤ 0.001); number signs (#) indicate statistically significant differences between time points of infection (24 vs 96 hpi) (#*p* ≤ 0.05, ##*p* ≤ 0.001)

At 24 hpi, a significantly higher gene expression of pro-inflammatory *il-12p35* and *cxcb2* as well as anti-inflammatory *arginase 1* and *2*, *il-10*, and *mmp9* was observed in the head kidney of E2-treated fish than in fish fed with control (non-E2) food (Figs. 7 and 8 and Table 2S).

At 96 hpi, gene expression of pro- and anti-inflammatory mediators was similar in E2- and non-E2-fed fish. In E2-treated fish, the expression of *il-12p35*, *cxcb2*, *arginase 1* and *2*, *il-10*, and *mmp9* was lower at 96 hpi than at 24 hpi (Figs. 7 and 8 and Table 2S).

At 24 hpi, E2 treatment did not affect the gene expression of pro- and anti-inflammatory mediators in the peritoneal leukocytes while at 96 hpi, it upregulated the expression of the *il-10* gene (Table 2 and 2S).

**Table 2:** In vivo effects of 17β-estradiol on the gene expression of pro-inflammatory mediators, CXC chemokines, and anti-inflammatory mediators in the peritoneal leukocytes (PTL).

Gene	24 hpi	96 hpi
*inos*	1.25 ± 0.24	1.39 ± 0.56
*il-1β*	1.24 ± 0.35	1.49 ± 0.36
*il-12p35*	1.23 ± 0.23	0.74 ± 0.17
*cxcl8_l2*	0.77 ± 0.14	1.51 ± 0.49
*cxcb2*	1.65 ± 0.56	1.37 ± 0.38
*arginase 1*	1.43 ± 0.64	1.78 ± 0.23
*arginase 2*	2.56 ± 0.76	1.22 ± 0.3
*il-10*	1.51 ± 0.32	1.95 ± 0.35*
*mmp9*	1.45 ± 0.29	1.6 ± 0.45

Fish were fed for 14 days with control food (non-E2) or food treated with 17β-estradiol (E2, 20 mg/kg food). On day 14 of E2 feeding, fish were injected i.p. with *A. salmonicida* (4 × 10^8^ bacteria in 250 μL PBS per fish). At 24 and 96 h post-infection (hpi), the peritoneal leukocytes were collected, and gene expression was measured. Changes in gene expression are shown as *x*-fold increase compared to control group (non-E2) and standardized for the housekeeping gene 40S ribosomal protein s11. Averages and S.E. (*n* = 7). Stars (*) indicate statistically significant differences in the gene expression in PTL derived from fish fed with control (non-E2) and E2-treated food at 24 or 96 hpi (*p ≤ 0.05)

At 96 hpi, E2 decreased in the liver expression of *inos* while it downregulated the liver expression of *crp2* at both at 24 and 96 hpi compared to control animals (Table 3 and 2S).

**Table 3:** In vivo effects of 17β-estradiol on the gene expression of pro- and anti-inflammatory mediators in the liver.

Gene	24 hpi	96 hpi
*inos*	1.36 ± 0.38	0.37 ± 0.16* **#**
*il-1β*	1.51 ± 0.61	1.34 ± 0.56
*arginase 1*	0.66 ± 0.34	2.2 ± 1.66
*arginase 2*	1.12 ± 0.41	2.24 ± 0.59
*il-10*	1.16 ± 0.45	2.39 ± 1.9
*c3*	0.77 ± 0.1	0.835 ± 0.4
*crp1*	0.913 ± 0.38	0.8 ± 0.19
*crp2*	0.48 ± 0.07*	0.18 ± 0.08**

Fish were fed for 14 days with control food (non-E2) or food treated with 17β-estradiol (E2, 20 mg/kg food). On day 14 of E2 feeding, fish were injected i.p. with *A. salmonicida* (4 × 10^8^ bacteria in 250 μL PBS per fish). At 24 and 96 h post-infection (hpi), the livers were collected, and gene expression was measured. Changes in gene expression are shown as *x*-fold increase compared to control group (non-E2) and standardized for the housekeeping gene 40S ribosomal protein s11. Averages and S.E. (*n* = 7). Stars (*) indicate statistically significant differences in the gene expression in the livers derived from fish fed with control (non-E2) and E2-treated food at 24 or 96 hpi (*p ≤ 0.05, **p ≤ 0.001). Number signs (#) indicate statistically significant differences between fish fed with E2-treated food in two time points (24 hpi vs 96 hpi) (#*p* ≤ 0.05)

#### Gene expression of estrogen receptors and Cyp19 aromatase

**Fig. 9:**
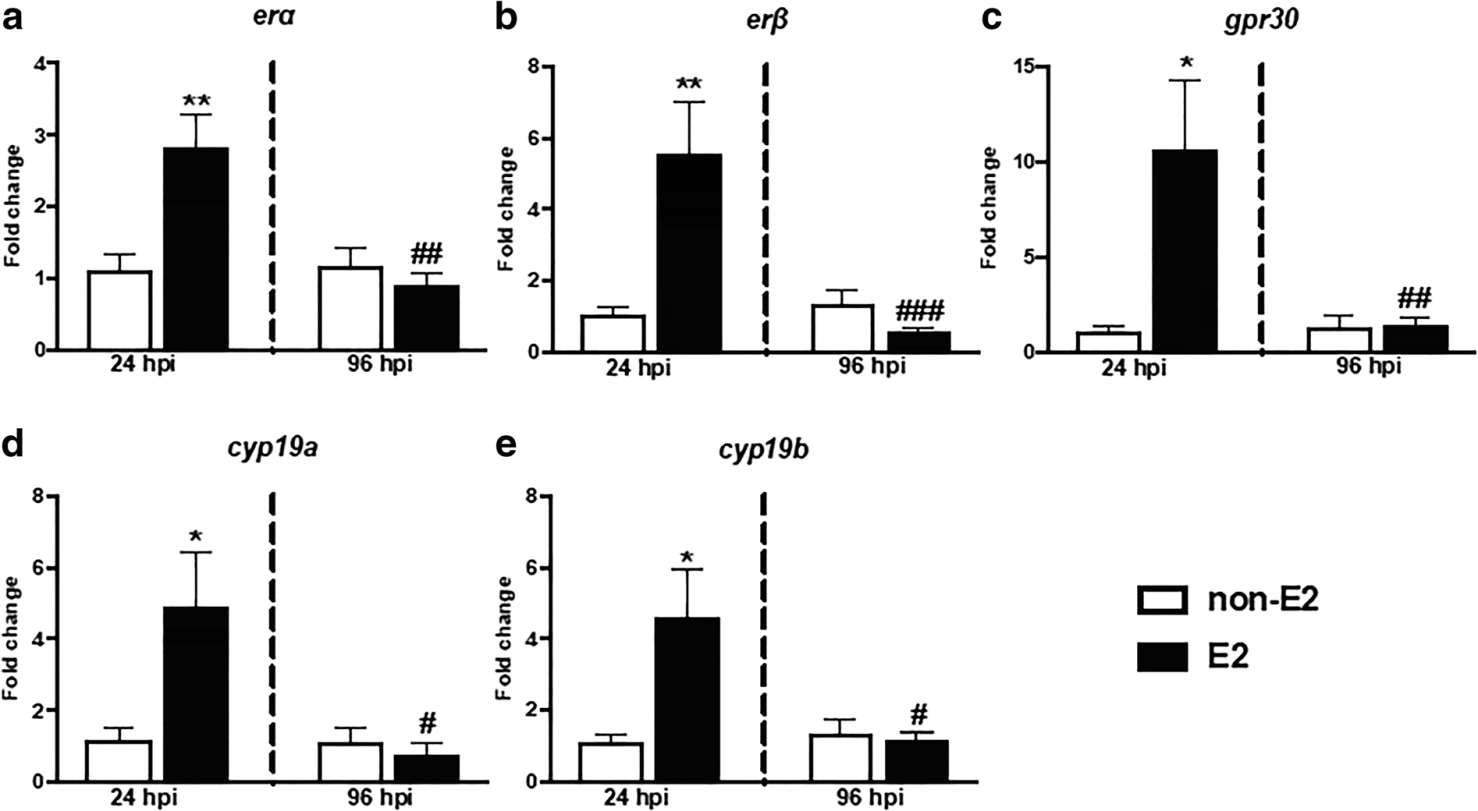
In vivo effects of 17β-estradiol on the gene expression of estrogen receptors (**a**–**c**) and aromatase CYP19 (**d**–**e**) in the head kidney. Fish were fed for 14 days with control food (non-E2) or food treated with 17β-estradiol (E2, 20 mg/kg food). On day 14 of E2 feeding, fish were injected i.p. with *A. salmonicida* (4 × 10^8^ bacteria in 250 μL PBS per fish). At 24 and 96 h post-infection (hpi), the head kidneys were collected, and gene expression was measured. Changes in gene expression are shown as *x*-fold increase compared to control group (non-E2) and standardized for the housekeeping gene 40S ribosomal protein s11. Averages and S.E. (*n* = 7). Stars (*) indicate statistically significant differences between control (CTR) and E2-treated animals (*p ≤ 0.05, **p ≤ 0.001); number signs (#) indicate statistically significant differences between time points of infection (24 vs 96 hpi) (#*p* ≤ 0.05, ##*p* ≤ 0.001, ###*p* ≤ 0.0001)

In the head kidney, E2 treatment upregulated the gene expression of the estrogen receptors (*erα*, *erβ*, *gpr30*) and of the aromatase (*cyp19a* and *cyp19b*) genes at 24 hpi (Fig. 9 and Table 2S). At the same point in the liver of E2-fed animals, the expression of the *gpr30* and *cyp19a* genes was lower than in animals fed with control food (Table 4 and Table 2S). Both at 24 and 96 hpi, E2 upregulated the expression of the *erα* gene in this organ.

**Table 4:** In vivo effects of 17β-estradiol on the gene expression of estrogen receptors and aromatase CYP19 in the peritoneal leukocytes (PTL) and in the liver (LV).

Gene	PTL		LV	
	24 hpi	96 hpi	24 hpi	96 hpi
*erα*	0.59 ± 0.08	1.47 ± 0.19**##**	12.4 ± 1.8***	10.1 ± 0.66***
*erβ*	0.74 ± 0.24	1 ± 0.17	0.88 ± 0.24	1.6 ± 0.32
*gpr30*	1.2 ± 0.15	1.33 ± 0.28	0.2 ± 0.1*	0.9 ± 0.25
*cyp19a*	2.27 ± 0.62	0.3 ± 0.1**##**	0.2 ± 0.09*	0.4 ± 0.9
*cyp19b*	0.59 ± 0.07	3.84 ± 1**##	0.52 ± 0.26	1.23 ± 0.21

Fish were fed for 14 days with control food (non-E2) or food treated with 17β-estradiol (E2, 20 mg/kg food). On day 14 of E2 feeding, fish were injected i.p. with *A. salmonicida* (4 × 10^8^ bacteria in 250 μL PBS per fish). At 24 and 96 h post-infection (hpi), the peritoneal leukocytes and livers were collected, and gene expression was measured. Changes in gene expression are shown as *x*-fold increase compared to control group (non-E2) and standardized for the housekeeping gene 40S ribosomal protein s11. Averages and S.E. (*n* = 7). Stars (*) indicate statistically significant differences in the gene expression in PTLs or livers derived from fish fed with control (non-E2) and E2-treated food at 24 or 96 hpi (*p ≤ 0.05, ***p ≤ 0.0001). Number signs (#) indicate statistically significant differences between fish fed with E2-treated food in two time points (24 hpi vs 96 hpi) (#*p* ≤ 0.05, ##*p* ≤ 0.001)

At 24 hpi, E2 treatment did not affect the gene expression of estrogen receptors and aromatase *cyp19a* and *cyp19b* in peritoneal leukocytes (Table 4 and 2S). An upregulation of the expression of *cyp19b* was observed in the peritoneal leukocytes (Table 4 and 2S).

## Discussion

In the present study, we focused on the in vitro and in vivo effects of 17β-estradiol on the innate immune response against bacterial infection in common carp.

### E2 in vitro decreases the LPS-induced activation of monocytes/macrophages

We found that in vitro in monocytes/macrophages E2 alone reduced the gene expression of the pro-inflammatory *inos*, *cxcb2*, and chemokine receptors *cxcr1–2.* Moreover, E2 in combination with LPS reduced the LPS-induced effects by decreasing the gene expression of pro-inflammatory mediators: *inos*, *il-1β*, *il-12p35*, *cxcb2*, and chemokine receptor *cxcr2.* These data suggest that during bacterial stimulation, E2 downregulates the cellular immune response. A similar pattern of response, indicating changes in IFN-γ-dependent cellular immune response, was also found in our recent experiment with carp monocytes/macrophages that were treated in vitro with pro-estrogenic endocrine-disrupting compounds (EDCs) such as 17α-ethynylestradiol (EE2) and 4-*tert*-octyphenol (Maciuszek et al., submitted).

Similarly, in vitro studies with the murine RAW264.7 macrophage cell line have shown that estradiol via ERα abolished the LPS-induced changes in early pro-inflammatory gene expression by avoiding intracellular NFκB transport without altering the IKK pathway (Ghisletti et al. 2005). Interestingly, E2 also reduced the LPS-induced expression of mediators that are involved in the resolution of inflammation, in angiogenesis, and in the process of wound healing/tissue repair such as *arginase 1* and *2*, *il-10*, *mmp9*, *cyr61*, *inhba*, and *tgm2* (Jaźwińska et al. 2007; Chadzinska et al. 2008a, b; Campbell et al. 2013; LeBert et al. 2015; Godwin et al. 2017; Hodgkinson et al. 2017; Hui et al. 2017; Costa and Power 2018). Previous studies on murine bone marrow-derived macrophages (BMDMs) also showed that pre-treatment with E2 decreased the LPS + INF-γ-induced *inos* expression whereas it did not affect the gene expression of *arginase 1* (Campbell et al. 2014). In the same study, E2 did not change *inos* and a*rginase 1* gene expression in IL-4-treated M2 macrophages. Moreover, the authors showed that the polarization of macrophages towards both the M1 and M2 phenotype passes via the ERα receptor and the lack of ERα in the macrophages prevents M2 polarization (Campbell et al. 2014). Similarly, Villa et al. (2015) found that in RAW 264.7 macrophages, E2 facilitated a progression of the inflammatory process towards the IL10-dependent “acquired deactivation” phenotype, which is responsible for tissue remodeling and the restoration of homeostatic conditions. This process takes place through regulation of the Socs3 and Stat3 signaling pathways. In contrast, Yang et al. (2012) found that, in vitro, E2 inhibited the alternative polarization of IL-4-stimulated tumor-associated ANA-1 macrophages. It reduced the arginase activity and the expression of CD206, and its action was associated with an inhibition of phosphorylation of the Jak1-Stat6 molecules and damage of the ERβ and ATPase interaction.

Also, in fish, data concerning the effects of E2 are often contradictory. For example, Yamaguchi et al. (2001) showed that E2 in vitro increased the phagocytic activity but did not change the NO and ROS production in carp macrophages. Conversely, in a goldfish macrophage cell line, E2 stimulation did not affect NO production but decreased phagocytosis (Wang and Belosevic 1995). Moreover, an immunomodulatory role of E2 was observed in gilthead seabream where it in vitro increased the expression of pro-inflammatory cytokines, chemokines, and toll-like receptor (TLR) genes as well as gene expression of tissue remodeling molecules, e.g., MMP13, in both naïve and *Vibrio anguillarum* genomic DNA (*Va*DNA)-stimulated macrophages. Furthermore, E2-treated seabream macrophages showed decreased phagocytosis, while no effects were observed on their ROS production or cell migration (Liarte et al. 2011b). In contrast, in adherent anterior leukocytes of channel catfish, bactericidal and ROS activity decreased upon E2 treatment (Iwanowicz et al. 2014). Moreover, recently, Paiola et al. (2019) found that in sea bass, E2 modulated redox biology and viability of myeloid cells in vitro in a dose- and time-dependent fashion. Also, cells of different origins (thymus, spleen, or head kidney leukocytes) showed a different response. In turn, our previous studies showed that E2 and agonists of the GPR30 receptor—G1 increased in vitro ROS production in PMA-stimulated carp monocytes/macrophages and this was reversed by pre-treatment of cells with wortmannin (inhibitor of PI3K) and G15 (GPR30 antagonist) but not by a selective antagonist of the nuclear estrogen receptor ICI 182:780 (Szwejser et al. 2017a). Similarly, E2 increased the respiratory burst of goldfish macrophages (Yin et al. 2007). In contrast to our studies, E2-induced changes in respiratory burst of channel catfish PBLs were blocked by the ER antagonist ICI 182:780 (Iwanowicz et al. 2014).

Interestingly, our results showed that E2 decreased the LPS-induced upregulation of pro-inflammatory mediators with an exception for *cxc8_*l1 expression, which in contrast was increased by E2 + LPS in a synergistic fashion. Previously, E2-induced enhancement of CXCL8 production was observed in unstimulated human monocyte-derived dendritic cells (Bengtsson et al. 2004) whereas in human peripheral blood monocytes, E2 attenuated the LPS-induced expression of CXCL8. This response was mediated through an estrogen receptor-dependent mechanism (Pioli et al. 2007). In fish, E2, alone or combined with bacterial DNA, was able to upregulate the expression of CXCL8 in gilthead seabream endothelial cells and macrophages (Liarte et al. 2011a, b)

It is intriguing that in the present study, E2 did not increase the in vitro expression of estrogen receptors or aromatase, whereas cell stimulation with LPS alone caused a decrease in *erα* and *erβ* expression. Similarly, LPS stimulation downregulated the expression of *erα1* and *2* in rainbow trout peripheral blood leukocytes (Shelley et al. 2012). In turn, in gilthead seabream macrophages, stimulation with VaDNA increased the gene expression of *erα*, *erβ1*, and *erβ2* (Liarte et al. 2011b). Our results support the hypothesis that in this way, the immune system changes the sensitivity to estradiol during inflammation. Moreover, Iwanowicz et al. (2014) found that ER gene expression was regulated in peripheral blood leukocytes of channel catfish following activation with concanavalin A and lipopolysaccharide. Also, in mammals, an LPS-induced downregulation of ER expression was observed in the RAW264.7 cell line, in microglial and in endothelin cells (Vegeto et al. 2004; Sierra et al. 2008; Holm et al. 2010).

E2-induced changes in the expression of estrogen receptors in mammalian and fish leukocytes are often contradictory. For example, Rastgar et al. (2019) found that long-term (3 days) incubation with E2 increased expression of *erα*, *erβ1*, and *erβ2* in cultured goldfish macrophages, while it was unaffected by 4-day incubation with E2 in rainbow trout peripheral blood leukocytes stimulated with LPS (Shelley et al. 2013)

### In vivo E2 treatment downregulates inflammatory response induced by *A. salmonicida* infection

Following the in vitro studies, where E2 suppressed the activity of LPS-stimulated monocytes/macrophages, we wanted to verify the effects of this estrogen on the anti-bacterial innate immune response in vivo. In this experiment, in animals fed with E2-treated food, a lower number of inflammatory phagocytes (monocytes/macrophages and neutrophilic granulocytes) was found in the peritoneum at 24 h post *A. salmonicida* i.p. injection. This suggests that in animals with increased E2 levels, the inflammatory reaction, manifested by leukocyte influx to the infection site, was weaker. Moreover, the anti-inflammatory action of E2 is corroborated by the slight but significant upregulation of the expression of anti-inflammatory *il-10* in peritoneal leukocytes at 96 hpi. Simultaneously, both at 24 and 96 hpi, E2 downregulated the gene expression of C-reactive protein in the liver, which supports the hypothesized anti-inflammatory action of E2. The elevated expression of both pro- (*il-12p35* and *cxcb2*) and anti-inflammatory (*arginase 1* and *2*, *il-10* and *mmp9*) at 24 hpi in the head kidney reflects the importance of this organ as main source of inflammatory leukocytes in teleost fish (Gruca et al. 1996; Chadzinska et al. 2008b). Therefore, differences in the gene expression in the head kidney between control and E2-treated fish can be a consequence of E2-induced changes in the leukocyte emigration from the head kidney to the peritoneum, the latter being the focus of inflammation. Although in the present experiment E2 treatment did not induce any fish mortality, we cannot exclude that harmful effects could be revealed at later stages of the infection. Previously, Wenger et al. (2011) found that E2 increased fish mortality and decreased the expression of hepatic complement components (*c3-1*, *c3-3*, and *factor-H*) in *Yersinia ruckeri*-infected rainbow trout. In turn, an in vivo study of juvenile sea bass exposed to E2 (20 ng/L) showed a reduction in the gene expression of pro-inflammatory cytokines (*il-1β*, *tnfα*, *il-6*) in the head kidney, compared to control animals and fish exposed to high E2 concentration (200 ng/L) (Seemann et al. 2013). Furthermore, studies in rainbow trout showed no E2-induced changes in the expression of *tnfα*, *il-1β*, and *cxcr4* compared to the control group that was not treated with E2 (Shelley et al. 2013) while exposure of largemouth bass to E2 reduced in the liver constitutive gene expression of antimicrobial hepcidin 1 and blocked induced during *Edwardsiella ictaluri* infection expression of hepcidin-2 gene (Robertson et al. 2009).

In our opinion, the most striking changes upon in vivo E2 treatment concerned upregulation of gene expression of mediators involved in the resolution of inflammation and in tissue repair (both arginases, *il-10* and *mmp9*) at 24 hpi. Similarly, in mammals, during zymosan-induced peritonitis, E2 treatment promoted an anti-inflammatory and pro-resolving phenotype in zymosan-induced peritoneal macrophages, which correlated with the induction of genes involved in macrophage alternative activation and with IL-10 expression in vivo. E2 also accelerated wound healing, while reducing the influx of leukocytes to the site of inflammation (Emmerson et al. 2009). Also, Campbell et al. (2010) found that the healing process is specifically dependent on estrogen receptor ERα.They observed that in mice with ERα gene disruption, wound healing and alternative polarization were delayed. On the other hand, exogenous administration of E2 to WT mice reduced the area of wound healing and increased wound re-epithelialization. Similar results were obtained by Routley and Ashcroft (2009), suggesting that lower levels of steroid hormones (estrogen or progesterone) cause polarization towards M1 (increased *tnfα* expression), while higher concentration of hormones shift polarization towards the M2 phenotype. Also, Ashcroft et al. (2003) found that estradiol accelerates wound healing by local inhibition of macrophage migration inhibitory factor (MIF).

So far, only limited studies, describing E2-induced changes in the expression of anti-inflammatory and wound healing markers, were published for teleost fish. For example, Seemann et al. (2013) found that estradiol did not affect the expression of *tgfβ* in the head kidney or change the level of TGFβ protein in the blood of juvenile sea bass. In gilthead seabream, estradiol increased the activity of MMP2 and MMP9 in testicle leukocytes (Chaves-Pozo et al. 2018). It also accelerated wound repair after scale removal in gilthead sea bream possibly via ERβs. In this study, the proteome of regenerating skin from E2-treated and control fish differed in keratin and collagen isoform expression (Ibarz et al. 2013). In contrast, E2-induced suppression of expression of multiple genes involved in wound healing, differentiation and tissue remodeling was found in lice-infected skin of Atlantic salmon. In this study E2 also significantly decreased the expression of the IL-4/13 genes, cytokines involved in M2 macrophage polarization (Krasnov et al. 2015).

The different in vitro and in vivo effects of E2 that we observed may suggest that during infection, E2 does not affect macrophages in the focus of inflammation only, but rather exerts indirect actions involving other cellular targets or macrophages in other organs/tissues. Most probably, E2-induced reduction of the number of peritoneal leukocytes is correlated to their impaired emigration from the head kidney. Therefore, as mentioned before, the changes in gene expression of pro- and anti-inflammatory mediators in the head kidney and in the peritoneum should be also scrutinized in the light of the changes in leukocyte composition.

Conflicting in vitro and in vivo results have been also reported from studies investigating the effects of estrogens on macrophage effector functions in mammals. For example, in striking contrast with in vitro observations, long-term in vivo exposure to estrogens was demonstrated to enhance the LPS-induced transcription of pro-inflammatory cytokines (IL-12 and TNF-α), by microglial cells through ERα-dependent mechanisms (Soucy et al. 2005). Furthermore, despite the confirmation of the anti-inflammatory effect of short-term in vitro exposure to E2 on murine resident peritoneal macrophages, Calippe et al. (2008) found contradictory effects in vivo*.* Chronic in vivo administration of E2 to ovariectomized female mice markedly increased the expression of numerous inflammatory cytokines and of *inos* in peritoneal macrophages in response to LPS activation ex vivo. The same group (Calippe et al. 2010) found that chronic E2 administration to ovariectomized mice significantly increased the expression of pro-inflammatory cytokines in thioglycolate-elicited macrophages, while disruption of the ERα gene in macrophages totally abolished the effect of E2 on the expression of inflammatory mediators by both resident and inflammatory peritoneal macrophages.

### Conclusions

Our results reveal that E2 can significantly change the anti-bacterial immune response in common carp. We found that estradiol reduces the expression of LPS-induced pro- and anti-inflammatory mediators in vitro, while in vivo, it decreased the inflammatory reaction. Therefore, we can conclude that estrogens modulate the inflammatory status of macrophages and other leukocytes and thus alters the ability to mount an effective immune response. This substantiates a significant role for this interplay in the outcome of inflammatory pathologies.

## Electronic supplementary material

Table 1S *In vitro* effects of 17β-estradiol on the gene expression of immune mediators, of estrogen receptors and of aromatase CYP19, in head kidney monocytes/macrophages. Cells were *in vitro* treated for 6 h with lipopolysaccharide (LPS, 30 μg/mL), 17β-estradiol (E2, 1 μM) or their combination (E2+LPS). Basal gene expressions were standardized for the housekeeping gene 40S ribosomal protein s11). Averages and S.E (n=4-5). Stars (*) indicate statistically significant differences between control (CTR) and treated cells (E2, LPS and E2+LPS) (*p≤0.05, **p≤0.001, ***p≤0.0001), number signs (#) indicate statistically significant differences between LPS-treated cells (LPS) and E2- or E2+LPS-treated cells (#p≤0.05, ##p≤0.001, ###p≤0.0001), ampersands (&) indicate statistically significant differences between E2- and E2+LPS treated cells (&p≤0.05, &&p≤0.001, &&&p≤0.0001). (DOCX 15 kb).

Table 2S *In vivo* effects of 17β-estradiol on the gene expression of immune mediators, of estrogen receptors and of aromatase CYP19, in the head kidney, peritoneal leukocytes (PTL) and liver. Fish were fed for 14 days with control food (non-E2) or food treated with 17β- estradiol (E2, 20 mg/kg food). On day 14 of E2 feeding, fish were injected i.p. with *A. salmonicida* (4 x 108 bacteria in 250 μL PBS per fish). At 24 and 96 h post-infection (hpi) the cells/organs were collected, and gene expression was measured. Basal gene expressions were standardized for the housekeeping gene 40S ribosomal protein s11. Averages and S.E (n=7). Stars (*) indicate statistically significant differences between control (CTR) and E2-treated animals (*p≤0.05, **p≤0.001, ***p≤0.0001). Number signs (#) indicate statistically significant differences between time points of infection (24 hpi vs 96 hpi) (#p≤0.05,##p≤0.001, ###p≤0.0001). (DOCX 17 kb).
