# Supplementary material for: 17β-Estradiol affects the innate immune response in common carp
Source: Fish Physiol Biochem. 2020 Jun 9;46(5):1775–94. doi: 10.1007/s10695-020-00827-3 (PMC7427712; doi:10.1007/s10695-020-00827-3)
Supplement: Supplementary file 1 — In vitro effects of 17β-estradiol on the gene expression of immune mediators, of estrogen receptors and of aromatase CYP19, in head kidney monocytes/macrophages. Cells were in vitro treated for 6 h with lipopolysaccharide (LPS, 30 μg/mL), 17β-estradiol (E2, 1 μM) or their combination (E2+LPS). Basal gene expressions were standardized for the housekeeping gene 40S ribosomal protein s11). Averages and S.E (n=4-5). Stars (*) indicate statistically significant differences between control (CTR) and treated cells (E2, LPS and E2+LPS) (*p≤0.05, **p≤0.001, ***p≤0.0001), number signs (#) indicate statistically significant differences between LPS-treated cells (LPS) and E2- or E2+LPS-treated cells (#p≤0.05, ##p≤0.001, ###p≤0.0001), ampersands (&) indicate statistically significant differences between E2- and E2+LPS treated cells (&p≤0.05, &&p≤0.001, &&&p≤0.0001). (DOCX 15 kb). [file 10695_2020_827_MOESM1_ESM.docx]

Table 1S.

| **gene** | **CTR** | **LPS** | **E2** | **E2+LPS** |
| --- | --- | --- | --- | --- |
| ***inos*** | 0.067±0.014 | 2.29±0.464 *** | 0.019±0.009 * ### | 0.41±0.031 *# & |
| ***il-1b*** | 0.4151±0.076 | 8.022±0.878 *** | 0.618±0.186 # | 3.582±1.386 * # |
| ***il-12p35*** | 0.0011±0.0002 | 0.0039±0.0008 *** | 0.0027±0.00004 * | 0.0007±0.0002 ## & |
| ***cxcl8_l1*** | 0.1176±0.02 | 0.3992±0.06* | 0.06±0.001 ## | 0.83±0.07***# &&& |
| ***cxcl8_l2*** | 0.06±0.02 | 0.15±0.02 * | 0.06±0.03 | 0.09±0.04 |
| ***cxcb1*** | 0.01±0.004 | 0.006±0.002 | 0.006±0.004 | 0.009±0.006 |
| ***cxcb2*** | 0.047±0.007 | 0.236±0.06 ** | 0.007±0.002 * ### | 0.02±0.007 ### |
| ***cxcr1*** | 0.385±0.08 | 0.276±0.07 | 0.12±0.02 * | 0.14±0.03 * |
| ***cxcr2*** | 0.03±0.005 | 0.03±0.005 | 0.008±0.002 *## | 0.007±0.001 *## |
| ***cxcr3*** | 0.04±0.007 | 0.03±0.005 | 0.02±0.004 | 0.009±0.002 * # |
| ***arginase 1*** | 0.0009±0.0002 | 0.004±0.001 * | 0.002±0.001 | 0.0009±0.0004 # |
| ***arginase 2*** | 0.1378±0.02 | 1.184±0.14 *** | 0.637±0.239 | 0.4872±0.191 # |
| ***il-10*** | 0.0079±0.001 | 0.019±0.002 ** | 0.007±0.002 # | 0.005±0.002 ### |
| ***mmp9*** | 1.146±0.11 | 1.208±0.13 | 0.289±0.07 * ## | 0.2553±0.07 ** ## |
| ***cyr61*** | 0.003±0.0009 | 0.017±0.01 * | 0.0006±0.0002 ## | 0.003±0.001 # |
| ***inhba*** | 0.0006±0.0001 | 0.002±0.0006 * | 0.0006±0.0001 # | 0.0004±0.0001 # |
| ***tgm2*** | 0.018±0.006 | 0.05±0.012 * | 0.019±0.009 | 0.024±0.005 |
| ***erα*** | 0.018±0.004 | 0.0082±0.001 * | 0.014±0.007 | 0.0037±0.001 * |
| ***erβ*** | 0.0013±0.0005 | 0.0003±0.0002 * | 0.0004±0.0002 | 0.0003±0.0001 * |
| ***gpr30*** | 0.0007±0.0003 | 0.0009±0.0003 | 0.0005±0.0002 | 0.0008±0.0006 |
| ***cyp19a*** | 0.0004±0.0002 | 0.0002±0.00007 | 0.0002±0.00009 | 0.0001±0.00001 |
| ***cyp19b*** | 0.0003±0.00006 | 0.0007±0.0003 | 0.0007±0.0003 | 0.0008±0.0007 |
