# Supplementary material for: 17β-Estradiol affects the innate immune response in common carp
Source: Fish Physiol Biochem. 2020 Jun 9;46(5):1775–94. doi: 10.1007/s10695-020-00827-3 (PMC7427712; doi:10.1007/s10695-020-00827-3)
Supplement: Supplementary file 2 — In vivo effects of 17β-estradiol on the gene expression of immune mediators, of estrogen receptors and of aromatase CYP19, in the head kidney, peritoneal leukocytes (PTL) and liver. Fish were fed for 14 days with control food (non-E2) or food treated with 17β- estradiol (E2, 20 mg/kg food). On day 14 of E2 feeding, fish were injected i.p. with A. salmonicida (4 x 10^8^ bacteria in 250 μL PBS per fish). At 24 and 96 h post-infection (hpi) the cells/organs were collected, and gene expression was measured. Basal gene expressions were standardized for the housekeeping gene 40S ribosomal protein s11. Averages and S.E (n=7). Stars (*) indicate statistically significant differences between control (CTR) and E2-treated animals (*p≤0.05, **p≤0.001, ***p≤0.0001). Number signs (#) indicate statistically significant differences between time points of infection (24 hpi vs 96 hpi) (#p≤0.05,##p≤0.001, ###p≤0.0001). (DOCX 17 kb). [file 10695_2020_827_MOESM2_ESM.docx]

Table 2S.

|  | **24 hpi** | | **96 hpi** | |
| --- | --- | --- | --- | --- |
| **gene** | **Non-E2** | **E2** | **Non-E2** | **E2** |
| **Head kidney** | | | | |
| ***inos*** | 0.193±0.032 | 0.216±0.091 | 0.017±0.006 # | 0.005±0.001 # |
| ***il-1β*** | 0.537±0.113 | 0.807±0.205 | 0.126±0.03 # | 0.121±0.019 ## |
| ***il-12p35*** | 0.001±0.0001 | 0.007±0.001*** | 0.001±0.0002 | 0.001±0.0005 ### |
| ***cxcl8_l2*** | 0.297±0.025 | 0.349±0.115 | 0.024±0.004 # | 0.02±0.005 ## |
| ***cxcb2*** | 0.016±0.004 | 0.078±0.019 ** | 0.021±0.004 | 0.03±0.007 # |
| ***arginase 1*** | 0.001±0.0003 | 0.003±0.0008 * | 0.001±0.0004 | 0.001±0.0003 # |
| ***arginase 2*** | 0.035±0.004 | 0.073±0.011 * | 0.031±0.008 | 0.043±0.008 # |
| ***il-10*** | 0.066±0.14 | 0.157±0.044 * | 0.012±0.002 | 0.017±0.005 ## |
| ***mmp9*** | 0.305±0.03 | 1.794±0.428 * | 1.922±0.587 | 1.295±0.221 |
| ***erα*** | 0.006±0.001 | 0.021±0.004 ** | 0.016±0.004 | 0.012±0.004 # |
| ***erβ*** | 0.003±0.001 | 0.017±0.006 * | 0.012±0.004 | 0.008±0.001 # |
| ***gpr30*** | 0.0004±0.0008 | 0.0051±0.002* | 0.001±0.0004 | 0.001±0.0006 # |
| ***cyp19a*** | 0.0005±0.0002 | 0.002±0.0009 * | 0.0004±0.0001 | 0.0005±0.0002 # |
| ***cyp19b*** | 0.0003±0.0002 | 0.002±0.001 * | 0.002±0.001 # | 0.0015±0.0006 |
| **PTL** | | | | |
| ***inos*** | 4.147±1.067 | 4.386±1.047 | 0.147±0.042 ## | 0.23±0.095 ## |
| ***il-1β*** | 10.221±2.42 | 8.936±2.662 | 0.626±0.092 ## | 1.218±0.409 # |
| ***il-12p35*** | 0.006±0.002 | 0.005±0.001 | 0.004±0.001 | 0.004±0.001 |
| ***cxcl8_l2*** | 2.977±0.631 | 2.013±0.610 | 0.326±0.058 ## | 0.442±0.144 # |
| ***cxcb2*** | 0.107±0.03 | 0.131±0.057 | 0.065±0.008 | 0.121±0.047 |
| ***arginase 1*** | 0.001±0.0002 | 0.001±0.0004 | 0.001±0.001 | 0.001±0.0005 |
| ***arginase 2*** | 0.519±0.146 | 1.172±0.377 * | 0.098±0.009# | 0.129±0.033 # |
| ***il-10*** | 0.044±0.006 | 0.078±0.03 | 0.033±0.006 | 0.069±0.01 |
| ***mmp9*** | 3.577±0.926 | 3.241±0.620 | 0.935±0.114 # | 1.315±0.45 # |
| ***erα*** | 0.024±0.006 | 0.008±0.001 * | 0.022±0.004 | 0.035±0.003 ## |
| ***erβ*** | 0.039±0.007 | 0.023±0.007 | 0.166±0.042 # | 0.134±0.028 ## |
| ***gpr30*** | 0.002±0.001 | 0.001±0.0001 | 0.003±0.001 | 0.002±0.001 |
| ***cyp19a*** | 0.001±0.0003 | 0.0015±0.0001 | 0.03±0.008 ## | 0.006±0.002 * |
| ***cyp19b*** | 0.001±0.0003 | 0.0004±0.0001 | 0.0005±0.0002 | 0.003±0.0006 ***### |
| **LIVER** | | | | |
| ***inos*** | 0.048±0.01 | 0.087±0.027 | 0.032±0.014 | 0.024±0.015 * |
| ***il-1β*** | 0.11±0.032 | 0.177±0.074 | 0.013±0.004 | 0.012±0.004 |
| ***arginase 1*** | 0.021±0.009 | 0.009±0.004 | 0.012±0.006 | 0.014±0.011 |
| ***arginase 2*** | 0.123±0.019 | 0.2±0.081 | 0.074±0.019 | 0.101±0.027 |
| ***il-10*** | 0.012±0.005 | 0.018±0.009 | 0.012±0.005 | 0.02±0.015 |
| ***erα*** | 0.063±0.027 | 0.414±0.082* | 0.063±0.026 | 0.848±0.19 ***# |
| ***erβ*** | 0.054±0.011 | 0.036±0.01 | 0.039±0.019 | 0.069±0.019 |
| ***gpr30*** | 0.016±0.004 | 0.003±0.001 | 0.017±0.016 | 0.003±0.001 |
| ***cyp19a*** | 0.021±0.005 | 0.004±0.002 ** | 0.003±0.002 # | 0.003±0.002 |
| ***cyp19b*** | 0.009±0.004 | 0.003±0.001 | 0.001±0.0004 | 0.003±0.001 |
| ***c3*** | 4.029±0.95 | 2.834±0.352 | 2.614±1.016 | 2.222±0.422 |
| ***crp1*** | 0.03±0.007 | 0.027±0.012 | 0.164±0.045 | 0.253±0.112 # |
| ***crp2*** | 0.013±0.004 | 0.003±0.001 | 0.044±0.013 | 0.017±0.008 |
| ***vitellogenin*** | 11.84±3.6 | 78.56±8.47 *** | 18.00±6.8 | 106.3±13.15 *** |
